# Heparin-Induced Thrombocytopenia in a Patient With Extensive Venous Thrombosis and Complex Comorbidities

**DOI:** 10.7759/cureus.95295

**Published:** 2025-10-24

**Authors:** Mohammed A Kassis, Mustafa H Adleh, Adel Mahmah, Rashed Oduibat, Ahmed M Saleh

**Affiliations:** 1 Department of Medicine, Ras Al Khaimah (RAK) Medical & Health Sciences University, Ras Al Khaimah, ARE; 2 Department of Medicine, Bahçeşehir University, Istanbul, TUR; 3 Department of Medicine, Alfaisal University, Riyadh, SAU; 4 Department of Internal Medicine, Wayne State University School of Medicine, Detroit, USA

**Keywords:** 4ts score, delayed onset hit type-2, hemorrhage, heparin-induced thrombocytopenia (hit), rbc alloimmunization

## Abstract

Heparin-induced thrombocytopenia (HIT) type 2 typically presents with thrombosis. Bleeding is uncommon and can obscure diagnosis. We describe a 69-year-old woman with chronic kidney disease who presented with progressive left-leg swelling. Initial evaluation included ultrasonography. However, an abrupt >50% platelet fall after recent heparin exposure raised clinical suspicion for HIT; therefore, serial 4Ts reassessments and an anti-platelet factor 4 (PF4)/heparin immunoassay were performed. Management included open thrombectomy, cautious anticoagulation, and transfusion minimization. Given hemorrhagic risk and renal impairment, apixaban was favored. Duplex ultrasound showed extensive acute deep vein thrombosis (DVT) in the left femoral system (common, profunda, and mid-femoral) and saphenofemoral junction, with chronic thrombi in the femoral and popliteal veins. During hospitalization, the patient developed recurrent severe anemia, large hematomas, and late thrombocytopenia with no bleeding source identified. Multiple erythrocyte alloantibodies were detected. Later during the hospital stay, serial 4Ts evolved to intermediate probability, and the anti-PF4 test was positive. Following appropriate measures, the patient was stabilized and subsequently discharged safely. Bleeding-dominant, alloimmunization-complicated courses can mask HIT. Serial 4Ts reassessment, careful laboratory interpretation, early transfusion-medicine input, and appropriate anticoagulant selection for renal impairment may reduce thrombotic and hemorrhagic risk.

## Introduction

Heparin-induced thrombocytopenia (HIT) is an uncommon, serious adverse reaction of heparin therapy, affecting around 2.6% of patients exposed to unfractionated heparin and approximately 0.2% of those receiving low-molecular-weight heparin [1,2]. Despite being uncommon, HIT can pose major risks for clotting events and death if not recognized early [1,3]. The underlying mechanism of HIT is immune reactivity, as antibodies develop against platelet factor 4 (PF4) bound to heparin, which in turn activate platelets, generate thrombin, and create a strong prothrombotic state [3]. What makes HIT particularly challenging is the paradox that patients present with low platelet counts while actually being at high risk for new clot formation.

The likelihood of HIT is higher in surgical and critically ill patients, especially when unfractionated heparin is used [2]. Clinicians are advised to combine careful bedside assessment with the 4Ts score-as this tool assigns 0-2 points for thrombocytopenia, timing of platelet fall, thrombosis or other sequelae, and other causes of thrombocytopenia (total 0-8; low 0-3, intermediate 4-5, and high 6-8)-to estimate pretest probability before ordering immunoassays [4]. A fall in platelets typically manifests within five to 10 days after exposure, and new thrombosis should raise suspicion [4]. Delayed diagnosis can cause serious complications, including limb loss or fatal thromboembolism [5].

Treatment is aimed at stopping all forms of heparin and quickly switching to a non-heparin anticoagulant, such as a direct thrombin inhibitor or a factor Xa inhibitor [6-8]. This step can be lifesaving but is complicated by the patient’s pre-existing comorbidities, renal function, and bleeding risks. However, not all cases follow the classic presentation, and features such as delayed onset or prominent bleeding can make the picture less clear [3,9]. Our case reflects this problem directly: recurrent bleeding, anemia, and alloimmunization masked the typical thrombotic features of HIT, underlining the need for repeated reassessment and close multidisciplinary collaboration.

## Case presentation

**Figure 1:**
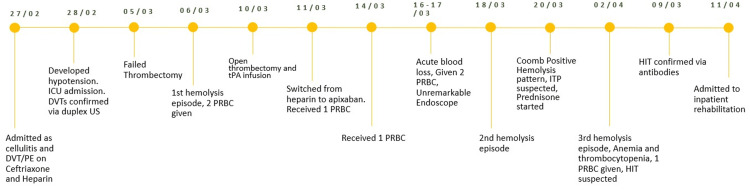
Timeline of patient management during hospitalization Hospital course timeline: heparin exposure, procedures (catheter-directed thrombectomy; open thrombectomy with intraprocedural tPA), hemoglobin/platelet nadirs, anti-PF4 ELISA result, transfusions, and anticoagulation decisions (with pauses). tPA: tissue plasminogen activator; PRBCs: packed red blood cells; HIT: heparin-induced thrombocytopenia; PF4: platelet factor 4; ELISA: enzyme-linked immunosorbent assay; DVT: deep vein thrombosis; PE: pulmonary embolism; US: ultrasound; ITP: immune thrombocytopenic purpura.

A 69-year-old woman with obesity (BMI 37), remote pulmonary embolism (PE), two prior left-leg deep vein thromboses (DVTs), an inferior vena cava filter, hypertension, stage three chronic kidney disease, prior left iliac vein angioplasties, and a chronic left lower-leg ulcer presented with 45 minutes of sharp chest pain radiating to the left shoulder and dyspnea. She also reported two days of worsening left-leg pain, swelling, and discoloration. She was hemodynamically stable. Physical examination showed a warm, tender, swollen left calf extending to the ankle with three ulcerations and dark purplish discoloration of the left thigh extending distally. A provisional diagnosis of DVT with possible PE was made, and treatment was initiated (Figure 1).

Initial testing showed normal ECG and troponin I levels. Due to chronic kidney disease, computed tomography (CT) pulmonary angiography was deferred; a ventilation/perfusion (V/Q) scan was also deferred because of chronic radiographic abnormalities (linear interstitial opacity in the left lower lung, suspected fibrosis/atelectasis). Laboratory markers revealed D-dimer 18,784 ng/mL (reference < 243 ng/mL), creatinine 2.2 mg/dL, leukocytosis 12.3 × 10⁹/L with neutrophilia 10.13 × 10⁹/L, and mild normocytic hypochromic anemia (hemoglobin 11.0 g/dL). Wound cultures were obtained from the ulcerated sites.

On hospital day (HD) 2, transient hypotension responded to fluids and holding antihypertensives. Bedside transthoracic echocardiography was normal. Left lower-extremity duplex ultrasound (US) demonstrated acute DVT in the common femoral, deep femoral, mid-femoral, saphenofemoral junction, and great and small saphenous veins, with chronic thrombi in the femoral and popliteal veins; arterial duplex US was normal. On HD 6, non-contrast CT abdomen/pelvis showed no overt bleeding.

Routine vitals and laboratory values are summarized in Table 1.

**Table 1:** Vitals and routine laboratory results with institutional reference ranges Reference ranges reflect our institutional laboratory. aPTT: activated partial thromboplastin time; PT: prothrombin time; INR: international normalized ratio; PF4: platelet factor 4; OD: optical density; BUN: blood urea nitrogen; AST: aspartate aminotransferase; ALT: alanine aminotransferase; HD: hospital day; HIT: heparin-induced thrombocytopenia.

Parameter	Admission HD 0	Severe anemia episode HD 6	Open thrombectomy HD 11	Positive anti-PF4 HD 37	Formal HIT diagnosis HD 41	Discharge HD 43	Units	Reference range
Temperature	97.5	97.8	98.0	96.9	97.1	97.8	Degree Fahrenheit	96.4-100.3
Heart rate	121	71	107	70	64	66	Beats/minute	60-100
Blood pressure (systolic/diastolic)	121/96	116/69	107/61	133/89	131/70	158/69	mmHg	90-160/50-90
Oxygen saturation	98%	98%	97%	98%	97%	98%	Percent	95-100
Platelet count	146	214	127	57	50	59	×10^9^/L	150-450
Hemoglobin	11.0	5.9	8.9	7.3	7.1	7.5	g/dL	12.0-15.5
White blood cells	12.3	8.1	7.5	6.9	5.3	5.6	×10^9^/L	4.5-10.5
aPTT	27.0	N/A	87.6	26.6	N/A	N/A	Seconds	26.8-34.8
PT	11.3	N/A	14.2	11.0	N/A	N/A	Seconds	9.4-14.2
INR	1.0	N/A	1.2	0.9	N/A	N/A	INR	0.9-1.3
Fibrinogen	N/A	N/A	326	160	N/A	N/A	mg/dL	234-434
D-dimer (fibrinogen equivalent units, FEU)	18,784	N/A	N/A	381	N/A	N/A	ng/mL	Less than 243
Anti-PF4 antibodies	N/A	N/A	N/A	0.75	N/A	N/A	OD	0.00-0.39
Haptoglobin	N/A	231	N/A	<8.0	<8.0	N/A	mg/dL	40-250
Direct antiglobulin test (Coombs)	Negative	N/A	N/A	Positive IgG	N/A	N/A		Positive/negative
Creatinine	2.17	1.36	1.12	1.28	N/A	N/A	mg/dL	0.57-1.1
BUN	41	34	19	31	N/A	N/A	mg/dL	10-20
AST	28	19	49	29	N/A	N/A	U/L	11-34
ALT	31	20	23	34	N/A	45	U/L	7-56
Reticulocyte count	N/A	N/A	124	228	234	221	×10⁹/L	44-106

Antimicrobials/wound care

On HD 0 (admission day), a continuous intravenous (IV) unfractionated heparin infusion was started for extensive DVT after a loading dose of 5,800 units. Empiric ceftriaxone 2 g IV once daily was initiated for five days to treat left-leg cellulitis. When wound cultures grew methicillin-resistant *Staphylococcus aureus* (MRSA), therapy was switched to vancomycin for 10 days, initially at 1,500 mg IV once daily, followed by dose adjustments to 750 mg IV once daily. The podiatry team performed surgical debridement and applied advanced dressings to optimize local wound control.

Anticoagulation and procedures

Initial duplex US confirmed acute DVT in the left common femoral, deep femoral, mid-femoral, saphenofemoral junction, and both great and small saphenous veins, with chronic thrombi in the femoral and popliteal segments; arterial duplex US was normal. Anticoagulation with continuous IV heparin was held intermittently when bleeding was suspected or procedures were planned. On HD 6, the patient underwent endovascular (peripheral) venogram with thrombectomy, which was unsuccessful and was followed by a severe anemia episode (hemoglobin 5.9 g/dL) accompanied by a moderate platelet decrease (from 214 × 10⁹/L to 141 × 10⁹/L). Heparin was stopped; two units of packed red blood cells (PRBCs) were transfused. On HD 7, non-contrast CT imaging ruled out internal bleeding, and heparin was resumed. On HD 11, an open thrombectomy of the left common femoral and proximal superficial veins with intraoperative tissue plasminogen activator (tPA) was successful. Due to the patient’s ongoing thrombotic risk, renal impairment, and the need to minimize major bleeding, anticoagulation was transitioned to apixaban 10 mg orally twice daily, which was started after surgery, then reduced to 5 mg orally twice daily as per standard acute venous thromboembolism (VTE) treatment. With the emergence of enlarging soft-tissue hematomas and evolving thrombocytopenia later in the course, apixaban was suspended on HD 35.

Bleeding/hemolysis evaluation

The patient experienced recurrent severe anemia requiring transfusion support. A comprehensive evaluation for bleeding and hemolysis was undertaken. Early direct antiglobulin testing (Coombs) was negative; haptoglobin was <8 mg/dL, lactate dehydrogenase (LDH) 991 U/L (reference 124-220 U/L), and total bilirubin 3.3 mg/dL with a predominantly direct fraction (indirect bilirubin 0.5 mg/dL). Esophagogastroduodenoscopy (EGD) and a nuclear medicine gastrointestinal bleed scan did not identify a bleeding source. Non-contrast CT of the chest/abdomen/pelvis demonstrated a 3.4 × 1.8 cm left inguinal fluid collection, favored to be a hematoma with trace pelvic fluid, and later imaging showed mild interval expansion. On repeat testing, Coombs became positive, and cross-matching identified multiple red blood cell (RBC) alloantibodies-anti-S, anti-E, anti-c, and anti-Jkᵇ-raising concern for a delayed hemolytic transfusion reaction. Therefore, a joint multidisciplinary decision to avoid further transfusions was made.

HIT testing and evolving probability

Clinical probability was reassessed serially using the 4Ts score in accordance with the American Society of Hematology (ASH) 2018 guidance. Early estimates were low (scores 3, then 1), but as new clinical data emerged-particularly the delayed and progressive platelet fall, with platelets declining from 214 × 10⁹/L to a nadir of 53 × 10⁹/L (>75% decrease, exceeding the ≥50% threshold supportive of HIT diagnosis [3,4])-probability increased to intermediate (score 4; see Table 2). On HD 37, anti-PF4 antibodies were positive (enzyme-linked immunosorbent assay (ELISA) optical density (OD) 0.75), and on HD 41, HIT was formally diagnosed in the context of the evolving thrombocytopenia and the patient’s heparin exposure.

**Table 2:** 4Ts score 4Ts score as per the American Society of Hematology 2018 guidelines [4], comparing all 3 major hemolysis episodes. ITP: immune thrombocytopenic purpura.

4Ts/episodes	1st hemolysis 3/6	2nd hemolysis 3/18	3rd hemolysis 4/2
Thrombocytopenia	0 (no platelet drop)	0	1
Timing of platelet decline	5-10 days 2	After 10 days 1	After 10 days 1
Thrombosis	0 (no new thrombosis)	0	0
Potential alternative cause of thrombocytopenia	1	0 ITP	2
Total score	3 (low score)	1 (low score)	4 (intermediate)

Supportive hematology care

Transfusion support consisted of PRBCs for severe anemia on HD 8 (two units), HD 12 (one unit), HD 15 (one unit), HD 17 (two units), and HD 34 (one unit). In view of the RBC alloantibodies, the decision shifted toward supportive therapy and avoidance of transfusion: erythropoietin 10,000 units subcutaneously (initiated during the hospitalization), IV ferric gluconate 125 mg on multiple days (clustered courses), and folic acid 1 mg orally once daily. Given an early differential that included immune thrombocytopenic purpura (ITP), prednisone 60 mg orally twice daily was started on HD 21 with close monitoring of platelet response and bleeding risk.

Response and recovery

Platelet counts remained near baseline early in the admission, then declined to a nadir of 53 × 10⁹/L later in the course, coinciding with expansion of soft-tissue hematomas and recurrent anemia. Hemoglobin dropped to 5.9 g/dL around HD 6-7 and to 6.7 g/dL on HD 20. Despite negative endoscopic and nuclear evaluations, the imaging-documented left inguinal hematoma and widespread ecchymoses accounted for much of the bleeding phenotype. After stopping heparin, careful use-and later withholding-of non-heparin anticoagulation, minimization of transfusions in the setting of alloimmunization, and supportive hematinic measures, platelets improved to 131 × 10⁹/L and hemoglobin stabilized at 8.6 g/dL. Consequently, the patient progressed from higher-acuity care to the rehabilitation ward in the same hospital, with improved pain control, better mobilization tolerance, and progressive physical therapy participation.

Outcome and follow-up

Once clinically stable with improving blood counts and no evidence of an active luminal bleed, the patient was discharged to inpatient rehabilitation, then home approximately one week later with continued improvement in exercise tolerance and wound healing. Outpatient hematology and vascular surgery follow-ups were arranged to reassess anticoagulation candidacy after the stabilization of hematomas and further platelet recovery. The post-discharge monitoring plan included weekly complete blood counts (CBCs) for four weeks, symptom surveillance for new thrombosis or bleeding, and wound-care review. No interval readmissions were documented in the available record.

## Discussion

This case shows how HIT can be diagnostically elusive when bleeding and transfusion-related factors dominate. Although typical HIT features include a platelet fall 5-10 days after heparin and a high thrombotic risk, this patient’s initial 4Ts scores were low to intermediate, the platelet nadir occurred later, and large hematomas with recurrent anemia obscured the usual prothrombotic phenotype. Nonetheless, the >75% platelet decrease strongly fulfilled one of the key diagnostic criteria for HIT, which was later supported by positive anti-PF4 antibodies [3-5].

Transfusion-related alloimmunization (anti-S, anti-E, anti-c, and anti-Jkᵇ) with a positive Coombs complicated the interpretation of hemolysis versus bleeding and increased the risk of delayed hemolytic transfusion reactions, further clouding diagnosis and management [10]. Given extensive DVT and prior thrombosis, apixaban was favored when anticoagulation was required because evidence shows lower major bleeding rates versus warfarin and efficacy for acute VTE; its pharmacokinetic profile also suits moderate renal impairment [6,8]. While ASH recommends functional assay confirmation of positive immunoassays to avoid false positives, such testing was not available in our case [4].

This experience highlights the value of case-based risk assessment: low initial scores should not preclude reconsideration as new data emerge, especially when thrombosis recurs or anemia evolves despite supportive measures. Clear documentation of heparin exposure timing, platelet nadir and recovery, and competing causes of thrombocytopenia can prevent premature diagnostic closure. In bleeding-dominant presentations, early engagement of transfusion medicine and careful selection of anticoagulants can mitigate both thrombotic and hemorrhagic hazards.

## Conclusions

HIT can be diagnostically challenging, particularly when it presents atypically with bleeding rather than thrombosis. In this patient, RBC alloimmunization created an overlapping hemolytic picture that masked HIT. This underscores the limitations of tools like the 4Ts and the need for improved initial screening to avoid missed cases with low early likelihood. Management required an early, multidisciplinary approach; transfusion medicine involvement was crucial to navigate alloimmunization and prevent delayed hemolytic reactions. Given the high hemorrhagic risk and renal insufficiency, careful anticoagulant selection was essential. A coordinated, team-based strategy can minimize both hemorrhagic and thrombosis-related complications.
